# The anomalous effect of COVID-19 pandemic restrictions on the duration of untreated psychosis

**DOI:** 10.1192/bjo.2024.813

**Published:** 2024-12-04

**Authors:** Jessica Nicholls-Mindlin, Hadar Hazan, Bin Zhou, Fangyong Li, Maria Ferrara, Nina Levine, Sarah Riley, Sneha Karmani, Walter S. Mathis, Matcheri S. Keshavan, Vinod H. Srihari

**Affiliations:** Medical Sciences Division, University of Oxford, Oxford, UK; and Translational and Clinical Research Institute, Newcastle University, Newcastle upon Tyne, UK; Program for Specialized Treatment Early in Psychosis (STEP), Yale University School of Medicine, New Haven, Connecticut, USA; Yale Centre for Analytical Sciences (YCAS), Yale School of Public Health, New Haven, Connecticut, USA; Program for Specialized Treatment Early in Psychosis (STEP), Yale University School of Medicine, New Haven, Connecticut, USA; and Institute of Psychiatry, Department of Neuroscience and Rehabilitation, University of Ferrrara, Ferrara, Italy; Beth Israel Deaconess Medical Center, Harvard Medical School, Boston, Massachusetts, USA

**Keywords:** Psychotic disorders/schizophrenia, COVID 19, duration of untreated psychosis, pathways to care, first-episode psychosis

## Abstract

We investigated the impact of COVID-19 restrictions on the duration of untreated psychosis (DUP). First-episode psychosis admissions (*n* = 101) to the STEP Clinic in Connecticut showed DUP reduction (*P* = 0.0015) during the pandemic, with the median reducing from 208 days pre-pandemic to 56 days in the early pandemic period, and subsequently increasing to 154 days (*P* = 0.0281). Time from psychosis onset to antipsychotic prescription decreased significantly in the pandemic (*P* = 0.0183), with the median falling from 117 to 35 days. This cohort study demonstrates an association between greater pandemic restrictions and marked DUP reduction, and provides insights for future early detection efforts.

In March 2020, COVID-19 was declared a global pandemic.^1^ With no vaccine available, restrictions on movement and gathering were used to slow transmission. Despite no evidence for a reduction in the incidence of psychosis, presentations to emergency psychiatric services declined in the first months of the pandemic.^1^ In Connecticut, USA, average monthly presentations to one such service decreased from 113 to 82 during the pandemic.^2^ In a New York City service, there was a 43% decline in emergency psychiatric presentations.^3^ This paralleled a 44% reduction in presentations to general emergency services in the USA.^4^ The severity of psychiatric presentations,^5^ and proportion admitted,^1^ was higher during the early pandemic period.

In February 2019, the clinic for Specialized Treatment Early in Psychosis (STEP) in New Haven, Connecticut, completed an early detection campaign called ‘Mindmap’.^6^ The campaign deployed public education (via social and mass media), community outreach and detailing of referral sources, and rapid access via a single referral number. A quasi-experimental trial^7^ demonstrated that Mindmap was associated with an increase in referrals and engagement, and a reduction in the duration of untreated psychosis (DUP), compared with a contemporaneous control site. Following trial completion, STEP continued to systematically measure delays in access to treatment following psychosis onset. The intrusion of the pandemic presented an opportunity for understanding any association between COVID-19 restrictions and DUP. The clinic measured overall delay between psychosis onset and admission, including the time from psychosis onset to first antipsychotic prescription, and the subsequent delay from antipsychotic prescription to STEP admission. We hypothesised that COVID-19-related restrictions would be associated with an increase in DUP.

## Method

### Setting and sampling

The STEP clinic accepted all individuals aged 16–35 years with primary non-affective psychosis and DUP <3 years, who were resident within a catchment of ten towns (population 400 000) around New Haven, Connecticut, USA. All consecutive admissions to STEP between 1 February 2019 and 21 March 2022 were included in this analysis.

### Exposure

The exposure was COVID-19-related restrictions. The pre-pandemic epoch was defined as the end of the early detection campaign (1 February 2019) until pandemic onset (14 March 2020), a time of no restrictions. The early pandemic epoch was the 6-month period that began on 15 March 2020, when stringent restrictions in Connecticut were announced, until 14 September 2020, when most restrictions were lifted.^8^ Subsequent admissions until 21 March 2022, a time of minor restrictions, were included in the late pandemic epoch, which finished 2 years after pandemic onset.

### Measures

DUP-Total was assessed as the time in days between psychosis onset and enrolment into STEP. Components of this overall measure of delay were DUP-Demand (time in days between psychosis onset and first antipsychotic use) and DUP-Supply (time in days between first antipsychotic use and enrolment into STEP). Psychosis onset was dated on the day when the ‘presence of psychotic syndrome’ criteria were met as per the Structure Interview for Psychosis Risk Syndromes.^9^

### Statistical analysis

Participant characteristics were compared with one-way analysis of variance (ANOVA), Kruskal–Wallis test for continuous variables and the chi-squared test or Fisher's exact test for categorical variables. The Wilcoxon rank-sum test was used for pairwise comparison between pandemic epochs because of the skew of DUP data distribution. Analyses were carried out with SAS version 9.4 for Windows (Cary, North Carolina, USA).

### Ethical approval

The authors assert that all procedures contributing to this work comply with the ethical standards of the relevant national and institutional committees on human experimentation and with the Helsinki Declaration of 1975, as revised in 2008. Verbal consent was witnessed and formally recorded as set out by a protocol approved by Yale University Human Investigation Committee (protocol number: 1310012846).

## Results

### Sample characteristics

Participants in the pre-pandemic and pandemic epochs were broadly comparable, except for a higher proportion of American citizens in the late pandemic epoch and more permanent residents in the early pandemic epoch; factors unlikely to affect access to care (Supplementary Table 1 available at https://doi.org/10.1192/bjo.2024.813). Missing data were minimal, except in household income, which most patients reported ignorance about. Personal income (as a proxy for socioeconomic class) was relatively complete and not significantly different. The sample was young and racially and ethnically diverse, reflecting local demography. The rate of admissions to STEP in the different epochs remained stable (Supplementary Table 2).

### DUP

**Fig. 1:**
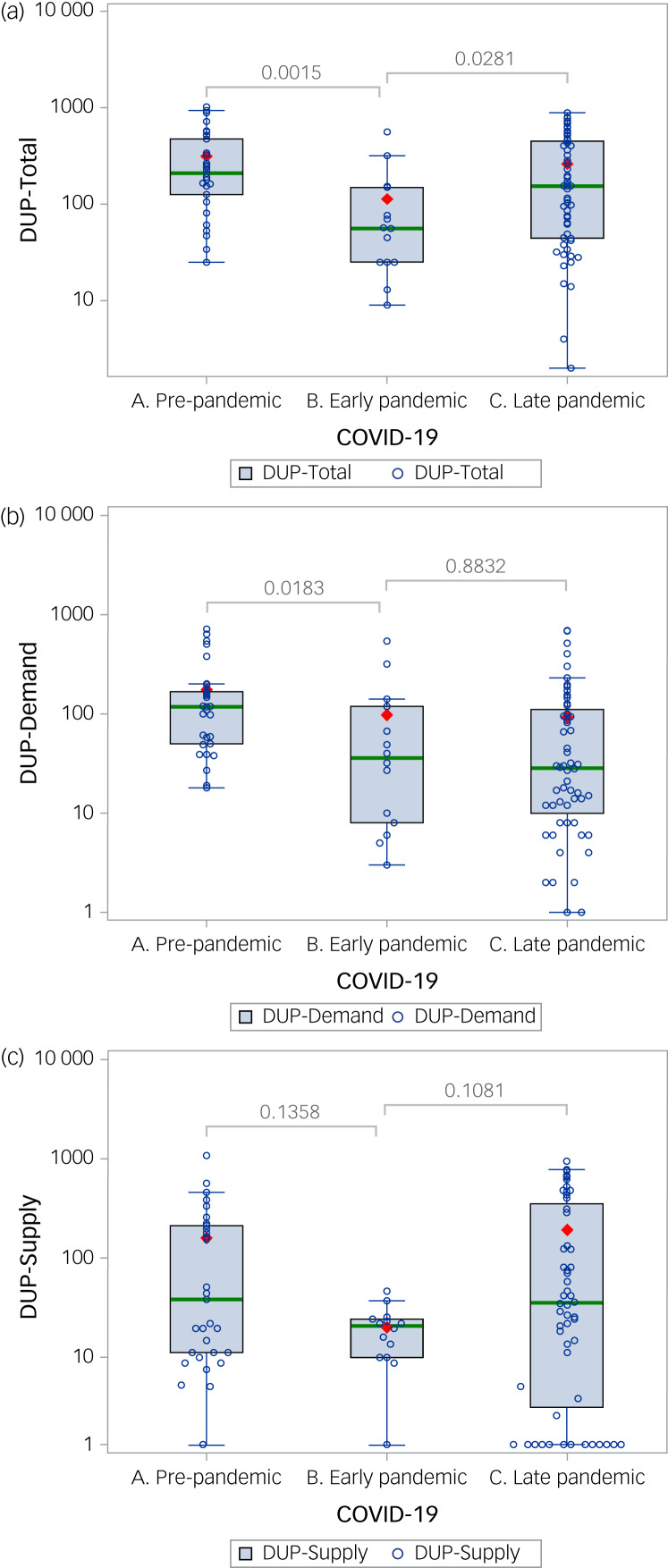
Box and whisker plots of DUP-Total, DUP-Demand and DUP-Supply in the pre, early and late pandemic epochs. Box and whisker plots of log (DUP) in the pre-, early and late pandemic epochs for (a) DUP-Total (b) DUP-Demand and (c) DUP-Supply. The green line represents the log (median), the box represents the log (25th and 75th) percentiles, the whiskers represent the maximum and minimum below the upper and lower fence, and the red diamond represents the log (mean). The results of pairwise comparisons of pre-and early pandemic, and of early and late pandemic, are reported. DUP, duration of untreated psychosis; DUP-Demand, time in days between psychosis onset and first antipsychotic use; DUP-Supply, time in days between first antipsychotic use and enrolment into the programme; DUP-Total, time in days between psychosis onset and enrolment into the programme.

Figures 1(a)–1(c) show box plots of DUP-Total, DUP-Demand and DUP-Supply, respectively, in the three epochs, with *P*-values for pairwise comparisons. Supplementary Table 3 shows descriptive statistics of the three epochs, including ranges. DUP-Total showed a significant reduction from pre-pandemic to early pandemic, with the median falling from 208 to 56 days (*P* = 0.0015). This drop appears to be largely accounted for by changes in DUP-Demand, which reduced significantly from a median of 117 to 35 days (*P* = 0.183). DUP-Supply did not change significantly, but followed the same pattern as DUP-Total, with a reduction followed by a rebound. DUP-Total significantly rose in the late pandemic compared with the early pandemic (*P* = 0.0281), although not reaching pre-pandemic levels.

## Summary and conclusions

The overall DUP decreased in the early compared with the pre-pandemic epoch. In the late-pandemic epoch, DUP-Demand persisted at this lower level, whereas DUP-Total increased. To the best of our knowledge, this is the first report to show a reduction in access delays for psychotic illness during the pandemic. We speculate that this anomalous DUP reduction was caused by earlier awareness by cohabitants of symptoms in the affected individual with whom they shared stringent restrictions against movement outside the home. This may have led to quicker help-seeking. Our prior analysis demonstrated that the single largest contributor to DUP in our region was the delay between psychosis onset and first-initiation of help-seeking, and that family members were key facilitators.^10^ Increasing family availability and lack of non-clinical options for help-seeking, such as school counselling resources, may have led to earlier clinical contact, thereby reducing delay. Additionally, reduced patient flow to normally crowded emergency services,^4^ with a proportional increase in presentations of psychotic disorder,^5^ may have allowed greater attention by emergency staff to the identification and management of psychotic symptoms. Both could have led to reductions in DUP-Demand. Since most patients in the catchment received their first antipsychotic via emergency and in-patient services,^10^ this reduction in DUP-Demand is unlikely to be explained by the increasing availability of telehealth services.

The reduction in DUP-Total during the most stringent restrictions and subsequent increase when these were loosened support a causal role for these restrictions in reducing delays to care. However, DUP-Demand remained low across the two pandemic epochs. This may be related to persistent family availability (e.g. work from home arrangements) to homebound patients, whereas decays in DUP-Supply may reflect return to pre-pandemic clinical workflows.

Some limitations should be noted. The small sample size in the early pandemic group (*n* = 14) increases the likelihood of chance findings. Nevertheless, this preliminary data from an established early detection service may be of interest to other first-episode services, which may observe similar patterns. Also, this data represents changes in DUP across one primarily urban and suburban region of the USA, which may not generalise to rural areas or areas with less malleable or structural sources of delay. Although families were dominant participants in help-seeking events during the trial and in the year after, we did not have complete data for this during the entire early and late pandemic phases. Finally, the early detection campaign may have had residual effects on pathways to care, and reduced scope for further DUP reduction in this population, although this would likely have reduced the effects observed in this analysis.

These findings shed light on patterns of presentation to a specialised psychosis service during a global health crisis. Periods of external threat have previously been associated with increased social cohesion;^11^ our results could support Durkheim's theory that major events create group integration,^12^ which may promote early detection. It highlights the potentially positive impact of greater attention by household members on reducing delays in care for new-onset psychotic disorders. The magnitude of the reduction in DUP in response to an environmental stimulus is broadly encouraging for early detection efforts that seek to engage observers in facilitating access to care.

## Supporting information

Nicholls-Mindlin et al. supplementary material Nicholls-Mindlin et al. supplementary material

## Data Availability

The data that support the findings of this study are available from the corresponding author, V.H.S., upon reasonable request.
